# The Care and Feeding of Infants

**Published:** 1884-10

**Authors:** 


THE CARE AND FEEDING OF INFANTS.

Such is the title of an interesting and useful little book, distributed *gratis* by the manufacturers of Mellin's Food. Of course, only the virtues of this food are lauded in the pamphlet, but all knowledge is useful, and this especially so, as many able physicians give this preparation a high place in their dietetic armamentarium. Dr. Eustace Smith, of London (Physician to the Children's Hospital, and author of *Wasting Diseases of Infants and Children*), says: Mellin's Food is by far the best of any with which I am acquainted. It seems to agree equally well with children whether they are healthy or diseased."

